# Neuroprotective and neuroregenerative effects of CRMP-5 on retinal ganglion cells in an experimental *in vivo* and *in vitro* model of glaucoma

**DOI:** 10.1371/journal.pone.0207190

**Published:** 2019-01-23

**Authors:** Jasmin Lauzi, Fabian Anders, Hanhan Liu, Norbert Pfeiffer, Franz Grus, Solon Thanos, Stefan Arnhold, Verena Prokosch

**Affiliations:** 1 Experimental Ophthalmology, Department of Ophthalmology, University Medical Center of the Johannes Gutenberg University Mainz, Mainz; 2 Department of Experimental Ophthalmology, School of Medicine, University of Münster, Münster, Germany; 3 Institute of Veterinary-Anatomy, -Histology and–Embryology, Justus-Liebig-University Gießen, Gießen, Germany; Bascom Palmer Eye Institute, UNITED STATES

## Abstract

**Purpose:**

To analyze the potential neuro-protective and neuro-regenerative effects of Collapsin-response-mediator-protein-5 (CRMP-5) on retinal ganglion cells (RGCs) using *in vitro* and *in vivo* animal models of glaucoma.

**Methods:**

Elevated intraocular pressure (IOP) was induced in adult female Sprague-Dawley (SD) rats by cauterization of three episcleral veins. Changes in CRMP-5 expression within the retinal proteome were analyzed via label-free mass spectrometry. *In vitro*, retinal explants were cultured under elevated pressure (60 mmHg) within a high-pressure incubation chamber with and without addition of different concentrations of CRMP-5 (4 μg/l, 200 μg/l and 400 μg/l). In addition, retinal explants were cultured under regenerative conditions with and without application of 200 μg/l CRMP-5 after performing an optic nerve crush (ONC). Thirdly, an antibody against Protein Kinase B (PKB) was added to examine the possible effects of CRMP-5. RGC count was performed. Number and length of the axons were determined and compared. To undermine a signal-transduction pathway via CRMP-5 and PKB microarray and immunohistochemistry were performed.

**Results:**

CRMP-5 was downregulated threefold in animals showing chronically elevated IOP. The addition of CRMP-5 to retinal culture significantly increased RGC numbers under pressure in a dose-dependent manner and increased and elongated outgrowing axons in retinal explants significantly which could be blocked by PKB. Especially the number of neurites longer than 400 μm significantly increased after application of CRMP-5. CRMP-5 as well as PKB were detected higher in the experimental than in the control group.

**Conclusion:**

CRMP-5 seems to play an important role in an animal model of glaucoma. Addition of CRMP-5 exerts neuro-protective and neuro-regenerative effects *in vitro*. This effect could be mediated via activation of PKB affecting intra-cellular apoptosis pathways.

## Introduction

Glaucoma, a neurodegenerative disorder, provokes an irreversible loss of retinal ganglion cells (RGCs) leading to optic nerve head excavation [1]. It is one of the leading causes of blindness worldwide [1, 2], with an increasing prevalence in people between the age of 40 and 80 [2, 3]. However, the pathophysiology of glaucoma remains unclear. Intraocular pressure (IOP) -lowering therapy, affecting the main risk factor of glaucoma, only retards the progression of the disease [4]. In contrast to the peripheral nervous system the central nervous system loses its capability of regeneration after injury [5, 6]. Thus, innovative neuro-protective and -regenerative therapies are urgently required for future glaucoma treatment [7, 8]. Thus, the idea of the study was to look for significantly changed key proteins within the course of glaucoma in an experimental animal model, which could alter the survival and regeneration of RGCs in glaucoma. By looking at important molecules in both neuro-regeneration and neuro-protection the collapsin response mediator protein (CRMP) was a promising match. The CRMP-family consists of five intracellular phosphoproteins (CRMP-1, CRMP-2, CRMP-3, CRMP-4 and CRMP-5) of a molecular size of 60–66 kDa. CRMPs are predominantly expressed in neurons and play important roles in axon formation and growth cone guidance and collapse. CRMPs have also been associated with neuronal degeneration. Among these CRMP-5 plays an important role in CNS development and differentiation [9, 10]. It is expressed in the embryonic and early postnatal CNS [11, 12], more precisely in post mitotic neuronal precursors [13]. CRMP-5 mediates growth cone guidance of neurites, neuronal polarity and longitudinal growth of axons [14, 15]. It has been shown that CRMP-5 increases Notch-receptor expression and thus activation of PKB (Akt) in tumor cell lines and glioblastoma stem cells [9]. PKB affects many intracellular pathways such as inhibition of apoptosis or expression of pro-survival genes [9, 16, 17]. In the adult CNS CRMP-5 expression reduces to a minimum, except for regions in the brain which run through processes of neurogenesis and in subpopulations of oligodendrocytes. However, in regenerating areas it is involved in development and cell survival of newly generated neurons [12–14, 18]. Based on these properties we suggest that CRMP-5 could protect glaucomatous damaged RGCs from apoptosis or rather enhance their regenerative capability by means of PKB. Thus, this study aimed first time at analyzing 1. the expression changes of CRMP-5 in an animal model of glaucoma, 2. its potential neuro-protective and neuro-regenerative effect and 3. its potential mechanism most probably via PKB.

## Material and methods

### Animals

For all experiments female Sprague-Dawley rats (n = 27) at the age of 8 weeks and a weight of 220–270 g were used. All experimental procedures were done in accordance with the ARVO Statement for the Use of Animals in Ophthalmic and Vision Research, and the guidelines of the Institutional Animal Care and Use Committee. An overview over the different experimental steps is provided in Table 1. The use of animals for research purposes was approved by the Landesuntersuchungsamt Rheinland-Pfalz (permission number: 14-1-085). All animals were accommodated at the Translational Animal Research Center of the Johannes-Gutenberg University Mainz with a day- and night-circle of 12 hours. Water and food were facilitated *ad libitum*. Surgical interventions were performed under general anesthesia using 0.02 ml medetomidine hydrochlorid (Dorbene, Zoetis, Germany) in combination with 0.08 ml ketamine (Ketamin Inresa, Inresa Arzneimittel GmbH, Germany) injected intramuscularly into the hamstrings. All animals were observed directly after each surgery and once a day for the next 72h. (Table 1)

**Table 1:** Overview of the different studies and their outcome.

Experiments	Proteomic changes	Neuroprotective effects of CRMP-5	Neuroregenerative effects of CRMP-5	Intracellular signal transduction
**Types**	Effect of glaucoma on retinal proteome	Effect of CRMP-5 on RGC- survival	Effect of CRMP-5 on neurite outgrowth of RGCs	to investigate intracellular absorption and interaction partners of CRMP-5
**Glaucoma model**	Episcleral vein oclusion *(in vivo)*	Untreated eyes, retinas in high pressure incubation chamber *(in vitro)*	Optic nerve crush *(in vivo)*, after 3 days retinal cell culture	High pressure chamber *(in vitro)*/ Optic nerve crush *(in vivo)*
**Stressing agent**	Elevated IOP	60 mmHg elevated hydrostatic pressure, oxidative stress	Damage of RGC-axons, oxidative stress	60 mmHg elevated hydrostatic pressure/ damage of RGC-axons, oxidative stress
**Time of follow-up**	7 weeks	2 days in culture	5 and 7 days in culture	2 days/ 7 days in culture
**Culture type**	Retinal explants	Retinal flatmounts	Retinal flatmounts	Retinal cross sections/ retinal flatmounts
**Assesment**	Label-free mass spectrometry	RGC count	Neuritecount and -measurement	Immunohistochemistry of samples of Neuroprotection and -regeneration studies
**Immuno-histochemistry**	/	Against BRN3A	Against β-III-tubulin	Against CRMP-5/ against PKB
**Concentrations**	/	200 μg/l CRMP-5	200 μg/l CRMP-5	anti-CRMP-5/ anti PKB
**Outcome**	Down regulation of CRMP-5	Neuroprotective—> CRMP-5 elevates RGC-survival	Neuroregenerative—> CRMP-5 promotes/elongates neurite outgrowth	CRMP-5 was taken up into RGCs and interacts with PKB to promote cell survival

### Thermic episcleral vein occlusion to induce elevated IOP

IOP was raised by thermic occlusion of three episcleral veins, reducing 50% of the venous outflow [19]. The intervention was performed unilaterally on the left eye (OS) (n = 7), while the right eye (OD) served as a contralateral control. IOP was measured prior to surgery to receive a baseline IOP and followed weekly over a total period of 7 weeks by a TonoLab rodent rebound tonometer (iCare, Finland) between 08:00 a.m. and 09:00 a.m. During the measurement animals were fixated through handholding and fully conscious. Ten sequenced readings were taken from the same area of the cornea and a mean value was built. Animals with unsteady IOP or without IOP elevation were excluded from the study.

### Quantitative proteomic measurements via label-free mass spectrometry

To analyze potential changes in the relative protein levels between experimental glaucoma- and contralateral control eyes, the retinal tissue was explanted from the enucleated eye balls and dissociated using liquid nitrogen and a mortar. Further breakdown of the retinal cells was performed chemically using 0.5% n-Dodecyl β-D-maltoside (Sigma Aldrich, USA) and mechanically using an ultrasonic bath and an ultrasonic wand. The tissue of each eye was treated and measured individually. Per sample, 80 μg of the retinal protein mix was loaded onto a NuPage Novex 12% Bis-Tris Protein Gel (Invitrogen, USA) and a SDS-PAGE was performed accordingly. The individual gel lanes were separated into 17 pieces, digested with sequencing grade trypsin and the peptide mix was extracted from the polyacrylamide gel and finally cleaned with C-18 ZipTips (Merck Millipore, USA). The peptide samples were analyzed with a capillary LC-ESI-MS system using a 50-minute linear gradient consisting of HPLC-grade water, acetonitrile, methanol and formic acid. The subsequent mass spectra were acquired using a LTQ OrbitrapXL (ThermoScientific, USA) with a resolution of 30,000 (m/z: 300–2,000). The obtained raw data was processed to the Maxquant proteome software (Max-Planck-Gesellschaft, Germany). Fixed modifications were limited to oxidation and acetylation, while the minimum peptide length was set to six. The false discovery rate (FDR) was adjusted to 1%. The LFQI intensities were further used to calculate the protein fold-changes and for statistical testing [20].

### Preparation and cultivation of retinal explants

Rats were euthanized through CO2 inhalation. Eyes were enucleated immediately post-mortem and transferred to a petri dish containing ice-cold betaisadona solution (Braunol, Braun, Germany) for 3 minutes and medium was then changed into ice cold sterile Hank's Balanced Salt Solution (HBSS; Gibco BRL, Eggenstein, Germany). The anterior segment of the eye was detached and the retina uncovered. Intact retina was separated from the optic cup and the vitreous body removed. Explants were placed like a shamrock with the ganglion cell layer up on Millipore filters (Millipore; Millicell; Cork, Ireland).

For studies on neuro-protection, retinal explants of untreated rats were cut equally into four pieces and transferred to petri dishes containing a hepes based medium with or without the addition of 200 μg/l/l CRMP-5 (CRMP-5 Polyclonal Antibody, Biossua) or an AB against PKB (Phospho-Akt (Ser473) (D9E) XP Rabbit mAb, Cell Signaling Technologies). Per experimental group the retinas of five different animals were used. Subsequently the petri dishes were placed into a high-pressure incubation chamber and incubated pressurized by 60 mmHg for 48 h.

For studies on neuro-regeneration, retinal explants of ONC-treated rats were cut equally into eight pieces by a tissue chopper (Mc Ilwain Tissue Chopper, Mickle Laboratory Engineering Co. Ltd.) three days after surgery. Upside down, these pieces were transferred into petriperm dishes, whose membranes have been coated with poly-D-lysine (Poly-D-Lysine solution, Merck, Germany) and Laminin (Mouse laminin-I, Sigma-Aldrich, USA) previously. Covered by a serum-free medium with or without the addition of 200 μg/l/l CRMP-5, the retinal explants were incubated for 7 days and half of the medium has been changed after 3 days in culture.

### Quantification of RGCs after studies on neuro-protection

For studies on neuro-protection RGCs were quantified by anti-BRN3A (Brn3a (C-20) goat polyclonal IgG, Santa Cruz Biotechnology, USA) immunohistostaining [21, 22]. Briefly the isolated retinas were fixed in 4%-formalin-solution (para-formaldehyde in Phosphate-buffered saline (PBS), pH 7.4), transmitted into 30% sucrose solution (sucrose in PBS) for 12 h and finally frozen in methylbutan for 3 seconds (Merck, Germany). The BRN3A-AB was diluted in 10% fetal calf-serum and incubated overnight at 4°C. Following RGCs were visualized with a fluorescent microscope (Axiophot Carl Zeiss, Germany) using 20-fold magnification. Ten pictures of every retinal piece were taken. Under usage of an ImageJ-macro RGC-count has been performed for every picture, an average value has been calculated and then extrapolated to RGCs/mm2.

### Unilateral ONC in combination with lens injury

ONC was conducted as previously described by Misantone et al. [20]. Briefly, five adult, female Sprague–Dawley rats were anesthetized as described previously. Local anesthetics of the cornea were assured by using topical drops of 0.4% oxybuprocaine (Novesine, Novartis, Germany). An incision was put to the upper eyelid parallel to the superior orbital edge. The optic nerve was exposed and clamped for 3 seconds using fine forceps considering that meninges were not clamped to guarantee a blood flow to the retina. From posterior of the bulb the lens was traumatized transsclerally with a glass capillary [21]. Terminally the incision was sutured and the eye was covered with antibiotic ointment (Ofloxacin, Floxal, Bausch+Lomb, Germany). Animals were euthanized 3 days after surgery.

### Quantification and measurement of neurite outgrowth after studies on neuro-regeneration

After 5 and 7 days in culture outgrowing neurites have been visualized and photographed natively with a binocular microscope (n = 10) with 40-fold magnification. On day 7 post explantationem (PE) neurites were identified immunohistologically by anti-β-III-Tubulin (Purified anti-Tubulin β 3, BioLegend). In brief the retinal explants with their neurites were fixed inside of the petriperm dishes in 4%-formalin-solution, blocked in 2% bovine serum albumin, 0.3% Triton-X and 5% goat serum for 60 minutes. The β-III-Tubulin-AB was diluted in blocking solution and incubated overnight at 4°C. Following neurites were visualized with a fluorescent microscope (Nikon Eclipse TS100, Germany) using 20-fold magnification. Under usage of Image J neurites have been counted and measured. Subsequently, mean values of the particular experimental groups were calculated and compared.

### Immunohistochemical staining of CRMP-5 and PKB

Retinal flatmounts, cultured for 48 h with and without the addition of CRMP-5, were paraffin fixed and cut into 10 μm thick cross sections subsequently. In a 60°C incubator the paraffin was melted overnight so that only the retinal cross sections themselves were left over on the object slides. By transferring the slides into Xylol (Xylene, AppliChem PanReac) and alcohol (Ethanol, Fisher Scientific; Ethanol 96% denatured with 1% MEK Technical grade PanReac, AppliChem) the cross sections got rehydrated and antigen-retrieval was performed with sodium citrate buffer (pH 6.0). CRMP-5 and PKB were visualized by anti-CRMP-5 (CRMP-5 Polyclonal Antibody, Biossua) and anti PKB (Phospho-Akt (Ser473) (D9E) XP Rabbit mAb, Cell Signaling Technologies). Briefly, the cross sections were blocked in 0.5% BSA, 0.1% Triton-X and 0.25% goat-serum for 60 minutes and incubated with either one or the other primary AB mentioned above overnight at 4°C. Following retinal cross sections were visualized with a fluorescent microscope (Axiophot Carl Zeiss, Germany) using 20-fold magnification. Neurites or more precisely flatmounts of the neuroregeneration studies were stained identically. Therefor retinal explants together with the outgrowing neurites were cut out of the petriperm dish membrane floor and transferred into 24-well plates. A negative control with the primary and secondary antibody alone served as the negative control.

### Protein analysis through antibody-microarray

CRMP-5-, PKB- and GAPDH- (as a loading control) Abs (GAPDH-AB, Sigma Aldrich, USA) (0.1 mg/mL) were spotted on a glass-nitrocellulose 16 multi-pad slide (Oncyte, Grace Bio-Labs, USA) with nine technical replicates per subarray using a non-contact array spotter (ciFLEXARRAYER 3, Scienion, Germany). Obeying the manufacturers protocol retinal protein mixes of the different experimental groups were labeled with Dylight 649 NHS Ester (Cy5) (ThermoFisher, USA). After 60 minutes of incubation the reaction was stopped by adding 100 μL of Tris-HCl for another 60 minutes. Simultaneously, the array slide was blocked for one hour with Grace Bio-Labs Super G blocking buffer (Sigma Aldrich, USA) and following washed with 0.5% Tween in PBS (3 times, 10 mins). According to the different experimental groups the Dylight-labeled protein mixes (10 μg in total) were loaded onto the particular subarrays and incubated for 2.5 hours. Subsequently the slide was washed three times with 0.5% Tween solved in PBS removing unbound proteins and dried in a SpeedVac-Concentrator for two minutes (Eppendorf, Germany). Terminatory, the fluorescence signals, emitted by bound labeled proteins, were scanned with a high-resolution confocal array scanner (Affymetrix 428, USA) at a gain of 10 dB and a line average of 10. With Imagene 5.5 (BioDiscovery Inc., USA) digitized signals were analyzed. (Table 1)

## Results

### Proteomic analysis of glaucomatous rat retinas

**Fig 1:**
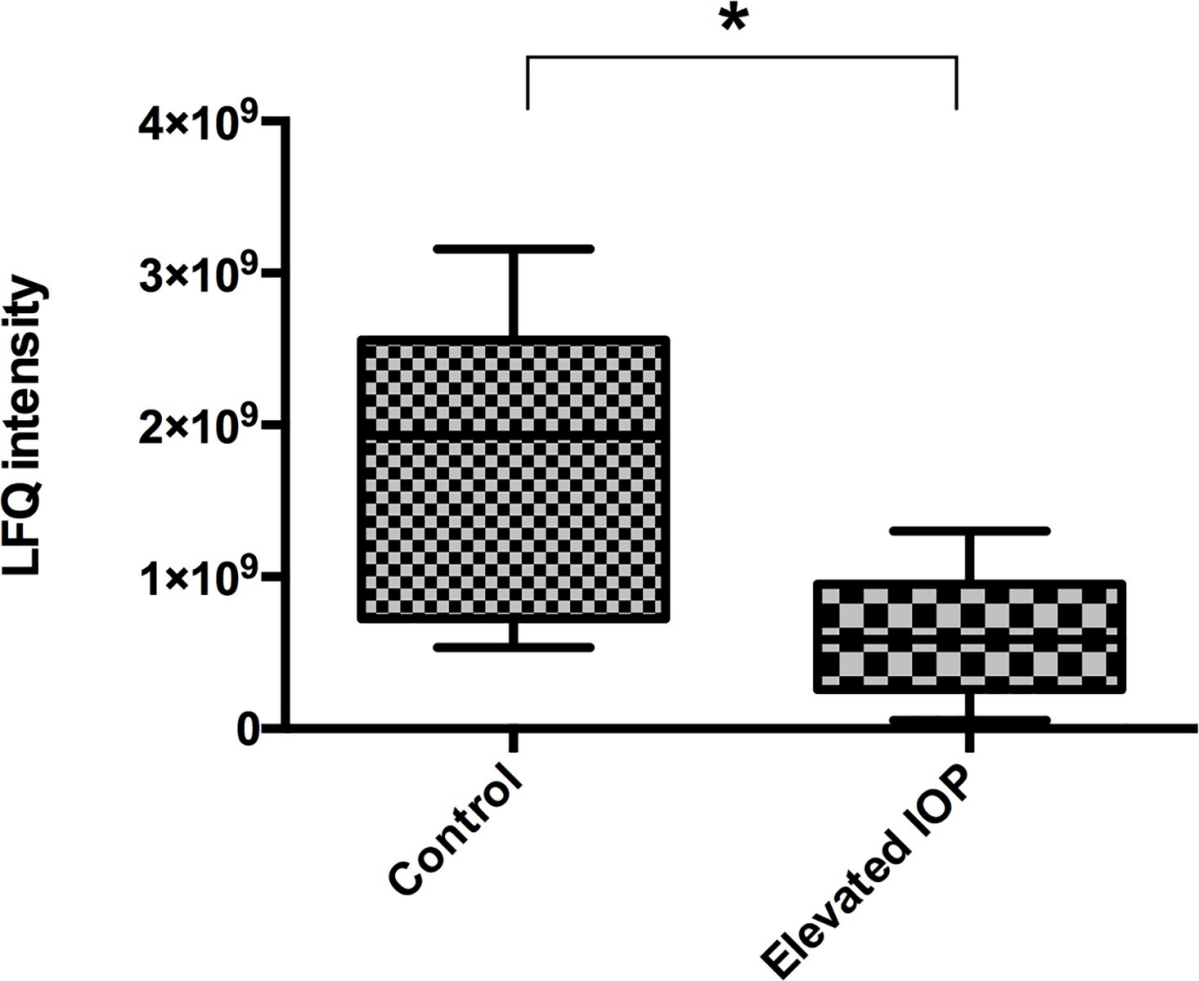
Mass-spectrometric quantification of CRMP-5 in the retinal proteome. CRMP-5 was detected significantly down-regulated in the experimental glaucoma group compared to the control group. Rat retinas were explanted after seven weeks of increased IOP and analysed via label-free mass spectrometry (p<0.0183, n = 7 per exp. group, ±SD, unpaired t-test).

Within the control group CRMP-5 was verified with a label-free quantification intensity (LFQI) of 1.93x10^9^. In comparison, in the experimental group showing chronically elevated IOP for seven weeks, CRMP-5 was detected significantly down regulated. Merely a LFQI of 0.59x10^9^ was verified. Hence CRMP-5 was existent three-fold down-regulated in the retinas suffering from experimental glaucoma (Fig 1).

### Neuroprotective effects of CRMP-5 and its interaction with PKB

**Fig 2:**
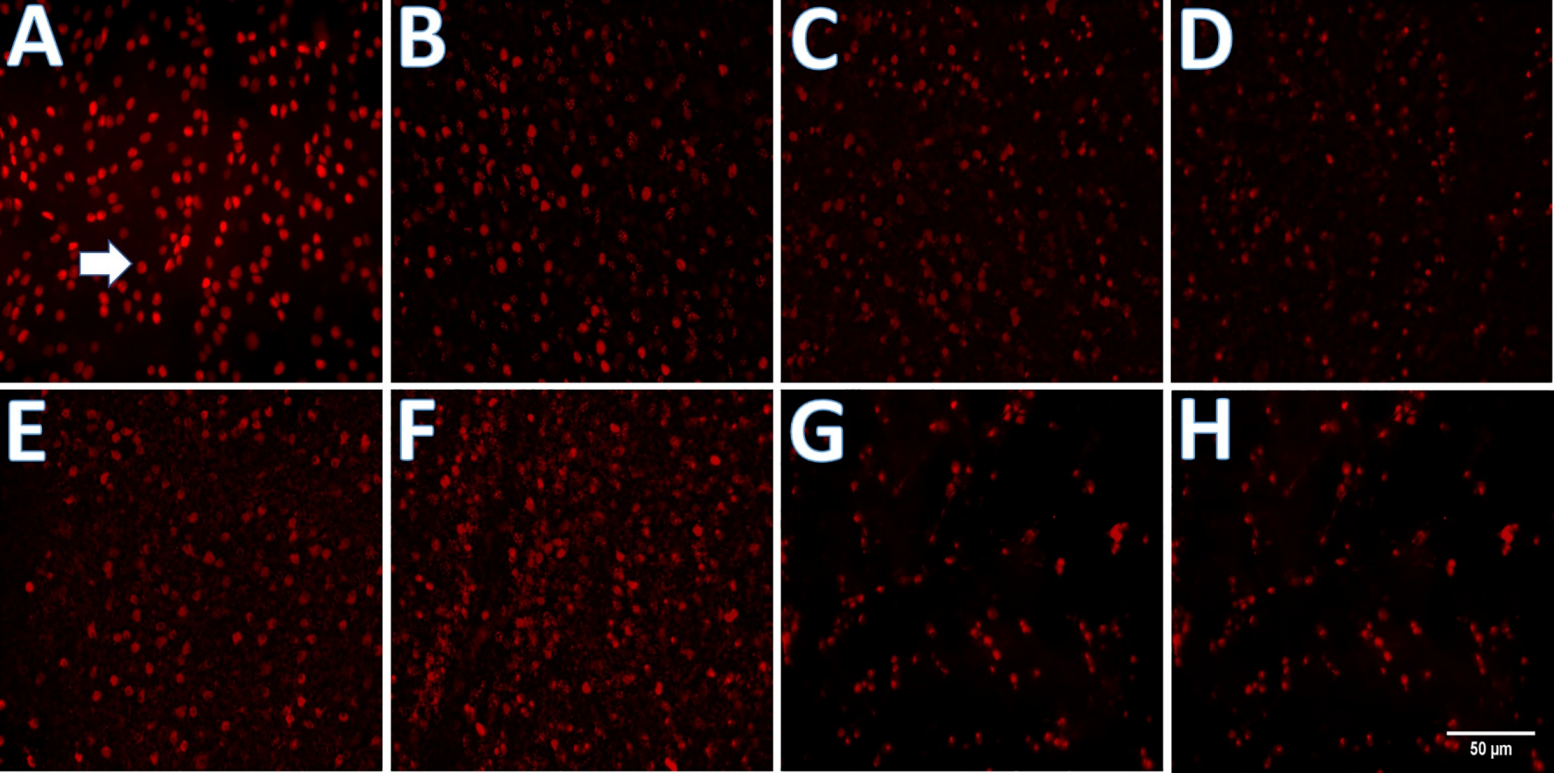
RGCs stained immunohistochemically against BRN3A. Retinas of adult rats were cultured in different experimental groups and subsequently fixed, embedded in paraffin and immunohistochemistry was performed. Vital RGCs are illustrated in red (arrow). Retinal explants were either not cultured at all (A), cultured for 24 h (B), cultured for 48 h (C), cultured under pressure of 60 mmHg for 48 h (D), cultured with the addition of 200 μg/l CRMP-5 for 48 h (E), cultured with the addition of 200 μg/l CRMP-5 under pressure of 60 mmHg for 48 h (F), cultured with the addition of 200 μg/l CRMP-5 under pressure of 60 mmHg and an AB against PKB for 48 h (G) or cultured with the addition of 200 μg/l denatured CRMP-5 under pressure of 60 mmHg for 48 h (H). Scale bar 50 um.

**Fig 3:**
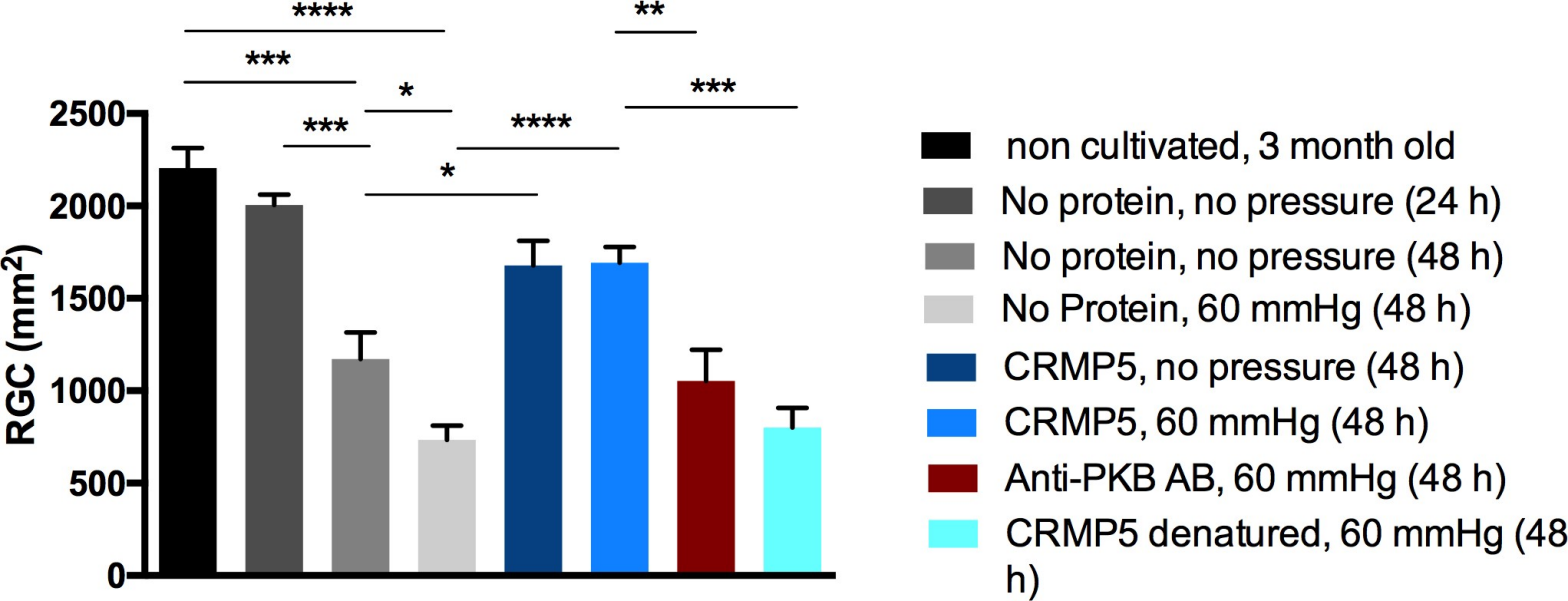
Quantification of neuroprotective effects of CRMP-5. Retinal flatmounts were incubated in a high-pressure incubation chamber, subsequently RGCs were stained against BRN3A and quantified. Different experimental groups are defined in uncultured retina (SEM = 108.4), cultured for 24 h (SEM = 56.24), cultured for 48 h (SEM = 143.5), cultured under pressure of 60 mmHg for 48 h (76,11), cultured with the addition of 200 μg/l CRMP-5 for 48 h (SEM = 134.5), cultured with the addition of 200 μg/l CRMP-5 under pressure for 48 h (SEM = 85.51), cultured with the addition of 200 μg/l CRMP-5 under pressure and an AB against PKB (SEM = 168) for 48 h and cultured with the addition of 200 μg/l denatured CRMP-5 under pressure for 48 h (SEM = 106.3). The addition of CRMP-5 intended a significant increase of surviving RGCs compared to the control group (n = 6, one-way ANOVA, p<0.05).

Cultured retinal explants were fixed after the predefined period of cultivation and predefined culture conditions. Subsequently, the retina pieces were stained immunohistochemically against BRN3A to examine morphological and numerical changes of RGCs (Fig 2). Fig 3 illustrates the number of quantifiable RGCs in the various experimental groups. Retinal explants were either not cultured at all (A), cultured for 24 h (B), cultured for 48 h (C), cultured under pressure of 60 mmHg for 48 h (D), cultured with the addition of 200 μg/l/l CRMP-5 for 48 h (E), cultured with the addition of 200 μg/l/l CRMP-5 under pressure for 48 h (F), cultured with the addition of 200 μg/l/l CRMP-5 under pressure and an AB against PKB for 48 h (G) or cultured with the addition of 200 μg/l/l denatured CRMP-5 under pressure for 48 h (H).

RGCs were quantified and mean values were compared. Fig 3 visualizes the conditions. Without cultivation an average amount of 2204 RGCs/mm^2^ was detected. After a cultivation period of 24 h the average amount of vital RGCs decreased to 2006 RGCs/mm^2^, whereas a cultivation of 48 h led to a decrease by half already (1171 RGCs/mm^2^). By additional pressure the amount decreased further to 735 RGCs/mm^2^. An application of 200 μg/l/l CRMP-5, prior to cultivation, increased the average amount of surviving RGCs cultured without (1677 RGCs/mm^2^) as well as with (1693 RGCs/mm^2^) additional pressure. The addition of an AB against PKB decreased the amount of RGCs again to 1054 RGCs/mm^2^. Denaturing of CRMP-5 also led to a decrease of RGCs (802 RGCs/mm^2^). In comparison to non-cultivated retinal explants cultivation for 48 h without (p<0.001) or with (p<0.0001) additional pressure showed a significant decrease in the amount of RGCs. Compared to an incubation for 24 h, an incubation for 48 h showed a significant decrease of vital RGCs (p<0.001). Furthermore, in comparison to an incubation of 48 h, additional pressure also caused a significant decrease of RGCs (p<0.05). Looking at flatmounts incubated without protein or pressure and at these ones incubated with protein and without pressure, it turned out that the addition of CRMP-5 led to a significantly higher amount of RGCs (p<0.05). This result was repeated in the comparison groups which were incubated with pressure and with or without the addition of CRMP-5. Here the application of CRMP-5 led to a highly significant increase in the amount of RGCs (p<0.0001). Compared to the retinal explants incubated with 200 μg/l/l CRMP-5 and with pressure of 60 mmHg an application of an AB against PKB and a denaturation of CRMP-5 caused a significant (p<0.01 and p<0.001) loss of vital RGCs.

### CRMP-5 increases neurite outgrowth

**Fig 4:**
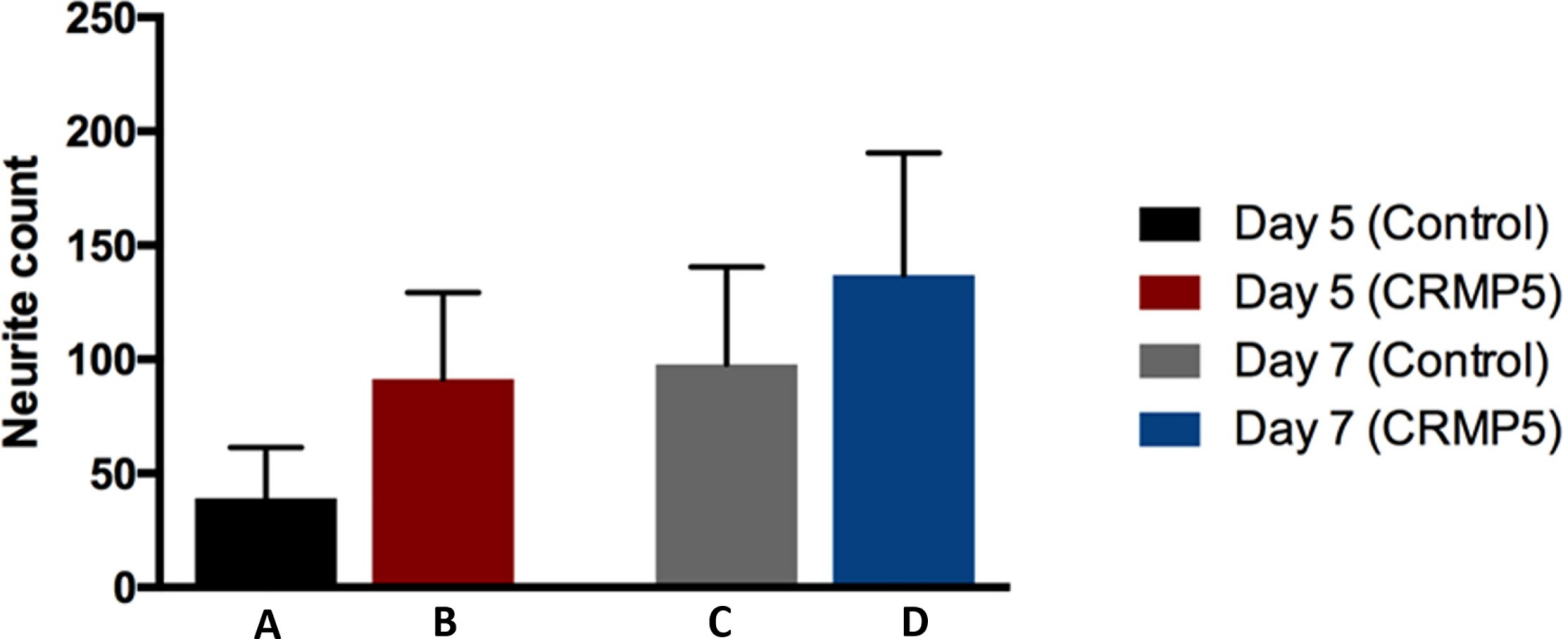
Quantification of outgrowing neurites in a neuroregenerative retinal organ culture. Retinal flatmounts were cultured under recovery-promoting conditions with (B,D) and without (A,C) the addition of CRMP-5. Outgrowing neurites were counted after five (A,B) and seven (C,D) days of cultivation. CRMP-5 caused an increased amount of neurite outgrowth in the experimental group compared to the control group (n = 5, ± SEM, parametric t-test).

To investigate neuroregenerative effects of CRMP-5 retinal flatmounts were cultured under regenerative conditions with and without an application of CRMP-5 after ONC. On day 5 *post explantationem* (PE) an average outgrowth of 39 neurites per retina-eighth (SEM = 22.15) was counted in the control group. In comparison 91 neurites per retina-eighth grew out after application of 200 μg/l CRMP-5 (SEM = 37.85). This describes a 2.3-fold increase of neurite outgrowth. On day 7 PE, compared to day 5 PE, the control group showed an increasing neurite outgrowth of 98 neurites per retina-eighth (SEM = 42.72). Again, there was a higher number of outgrowing neurites noticeable after an application of CRMP-5 (137 neurites per retina-eighth, SEM = 53.56). This describes an increase of 40% compared to the control group. However, these results did not show a significance (Fig 4).

**Fig 5:**
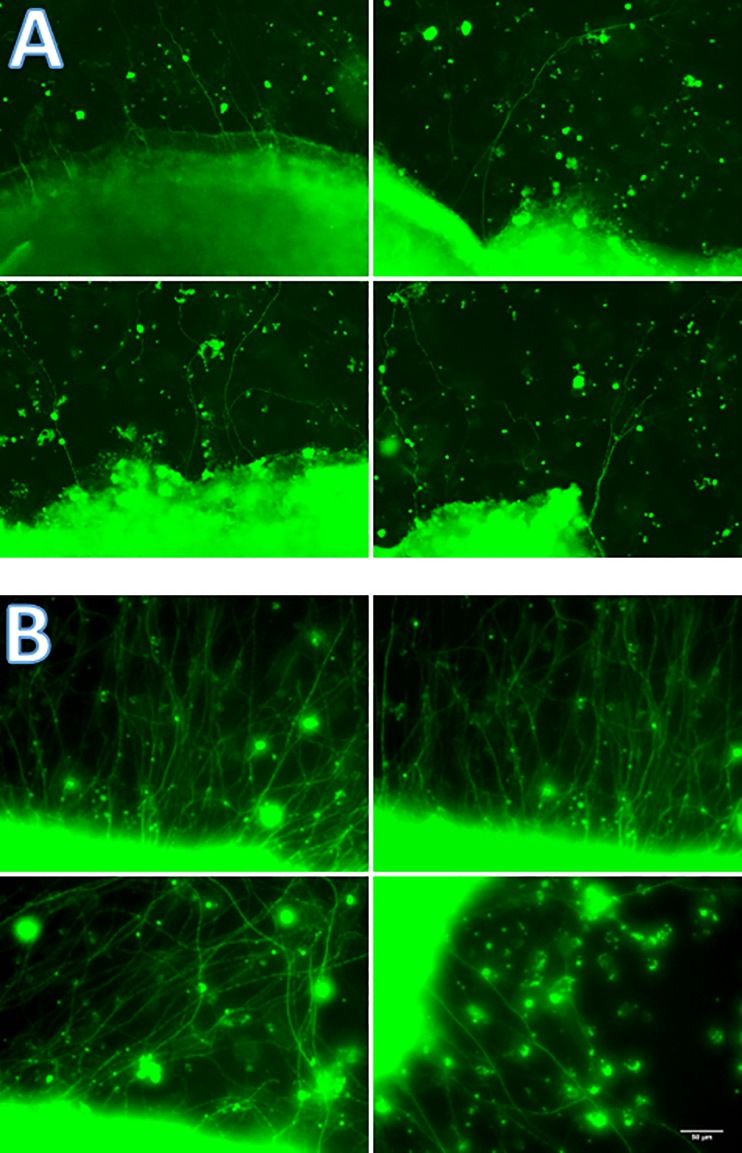
Immunohistochemical staining against neuron-specific β-III-Tubulin. After seven days of cultivation in serum-free medium the control group cultivated without CRMP-5 (A) and the group cultivated with the addition of CRMP-5 (B) were stained immunohistochemically against neuron-specific β-III-Tubulin. The CRMP-5 group was hallmarked by an increased neurite outgrowth. Scale bar 50 μm.

After 7 days of cultivation the neuronal structures of the organ culture were stained against β-III-Tubulin. Fig 5A illustrates outgrowing neurites of the control group. In contrast Fig 5B demonstrates outgrowing neurites of the retina pieces incubated with the addition of CRMP-5. Compared to the control group an immense increase in neurite outgrowth is audible. Cell processes are presented in FITC channel and thus in green.

### CRMP-5 elongates outgrowing neurites

**Fig 6:**
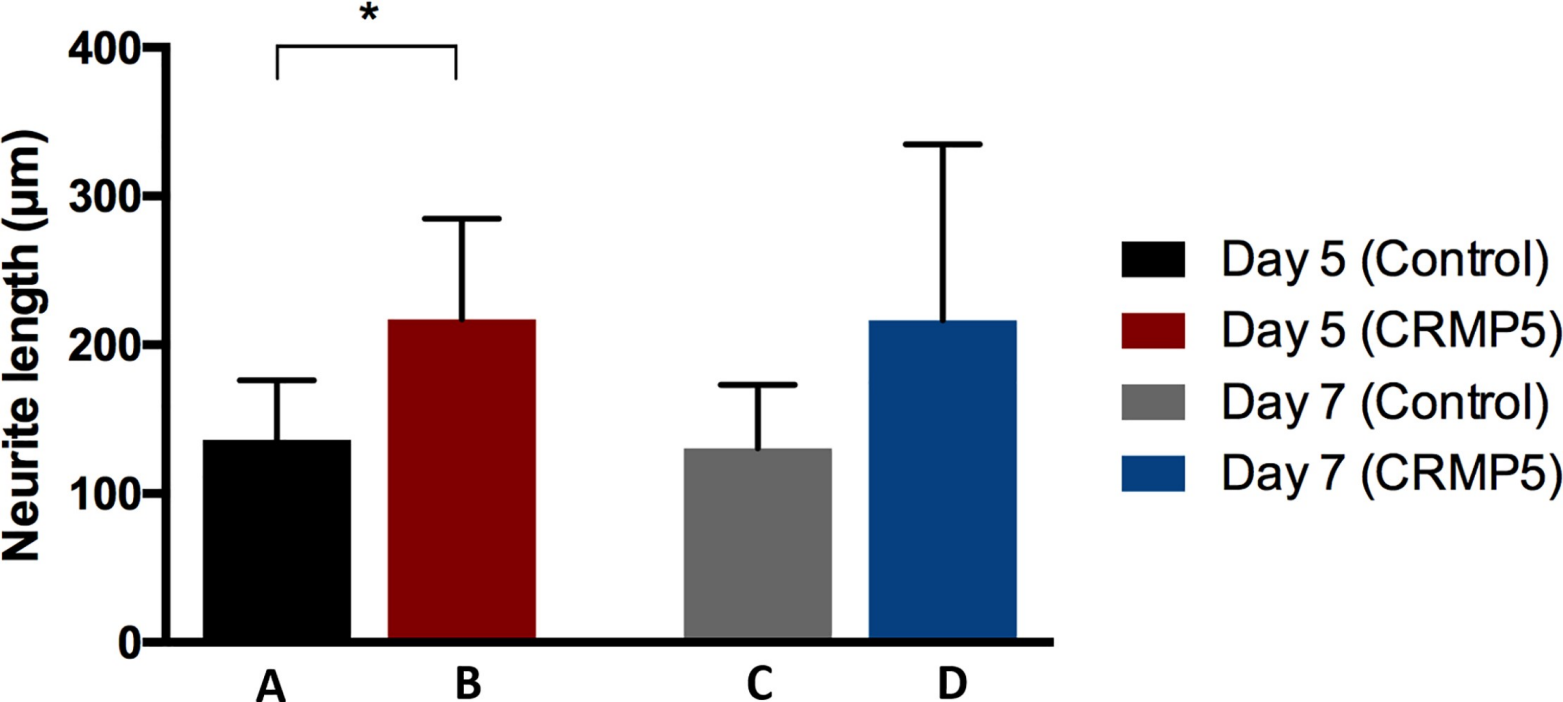
Length development of outgrowing neurites. Outgrowing neurites were measured. Mean length values of outgrowing neurites after five days of cultivation (A), after seven days of cultivation (B), after five days of cultivation with the addition of CRMP-5 (C) and after seven days of cultivation with the addition of CRMP-5 (D) were built. CRMP-5 caused a significant elongation compared to the control group (n = 5, p< 0.05, ± SD, parametric t-test).

For length detection, photographed outgrowing neurites were measured and mean values were built within the particular experimental groups. 5 days PE an average elongation growth of 136 μm was detected within the control group (SD = 40.16). An application of 200 μg/l CRMP-5 prolonged outgrowing neurites to an average length of 217 μm (SD = 67.53). This describes an average 1.6-fold elongation of neurites. 7 days PE an average neurite length of 131 μm was measured within the control group (SD = 42.6). Again, neurites lengthened 1.6-fold after the addition of CRMP-5 to an average length of 216 μm (SD = 118.1). Statistical analysis by a parametric t-test showed a significant extension of the neurites on day 5 PE through the application of CRMP-5 compared to the control group (p<0.05) (Fig 6).

**Fig 7:**
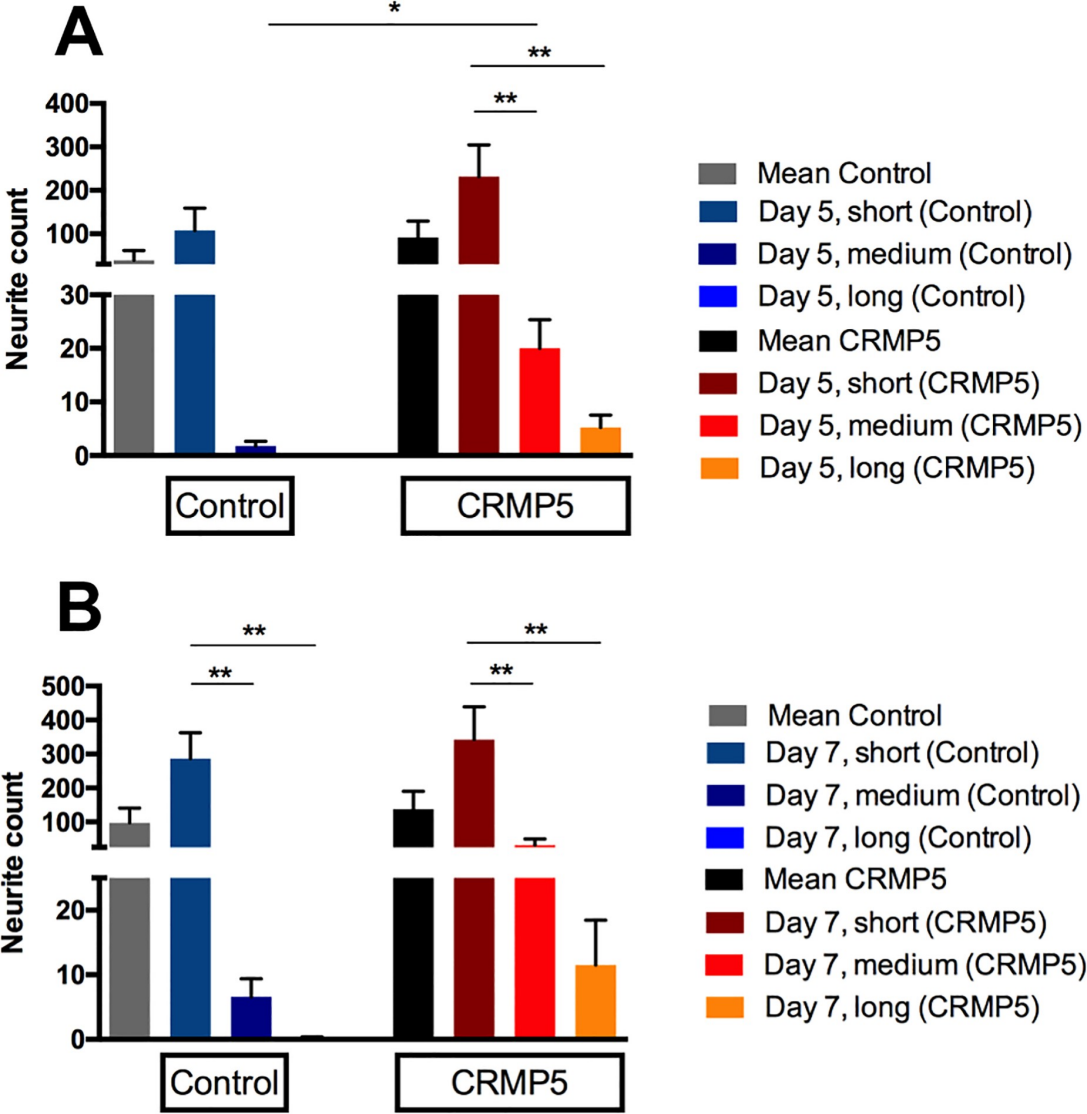
Classification of neurite length development. To draw a qualitative comparison of neurite length development between the experimental and the control group the measured neurites were classified into three groups (short < 400 μm, medium 400–800 μm, long > 800 μm). CRMP-5 caused a significantly increased outgrowth of medium neurites (n = 10, p < 0.05, ± SEM, one-way ANOVA, parametric t-test).

For detailed examination of neurite elongation outgrowing neurites were classified into three groups (short < 400μm, medium 400–800μm, long > 800μm). 5 days PE within the control group an average amount of 39 outgrowing neurits per retina eighth was counted (SEM = 22.15). With an average amount of 108, the breakdown of raw data clearly showed a dominance of short neurites (SEM = 51.27). No growth of long neurites was present and simply an average of two medium neurites was detected per retina eighth (SEM = 0.86). In opposition to that 91 neurites per retina eighth grew out in the CRMP-5 group (SEM = 37.85). Again, the breakdown of the raw data showed with an average of 232 a dominance of short neurites (SEM = 72.95). Besides that, a mean of 20 medium (SEM = 5.36) and 5 long (SEM = 2.29) neurites per retina eighth were detected. Statistical analysis by an unpaired t-test showed a significantly higher number of medium neurites growing out after the addition of CRMP-5 (p<0.05) (Fig 7A).

7 days PE a mean value of 91 neurites per retina eighth was counted within the control group (SEM = 42.72). Compared to neurite outgrowth on day 5 PE the amount increased 2.5-fold. The breakdown of raw data again showed a dominance of short outgrowing neurites with an average amount of 287 (SEM = 76.07). Simply 7 medium neurites (SEM = 2.77) and 0.2 long neurites (SEM = 0.2) expanded per retina eighth. In contrast an average of 137 neurites per retina piece was detected after the addition of CRMP-5 (SEM = 53.56). Hence the average amount of outgrowing neurites within the experimental group increased 1.5-fold compared to the outgrowth five days PE. In this case the breakdown of raw data demonstrated, with an average amount of 343, a distinct dominance of short neurites (SEM = 96) but besides that 31 medium (SEM = 18.32) and 11.5 long (SEM = 6.96) neurites per retina eighth were observed. Within the control group as well as within the experimental group statistical analysis by a one-way Anova-test established a significant decrease in the amount of outgrowing medium and long neurites compared to short ones (p<0.01) (Fig 7B)

### Microarray analysis of rat retinas

**Fig 8:**
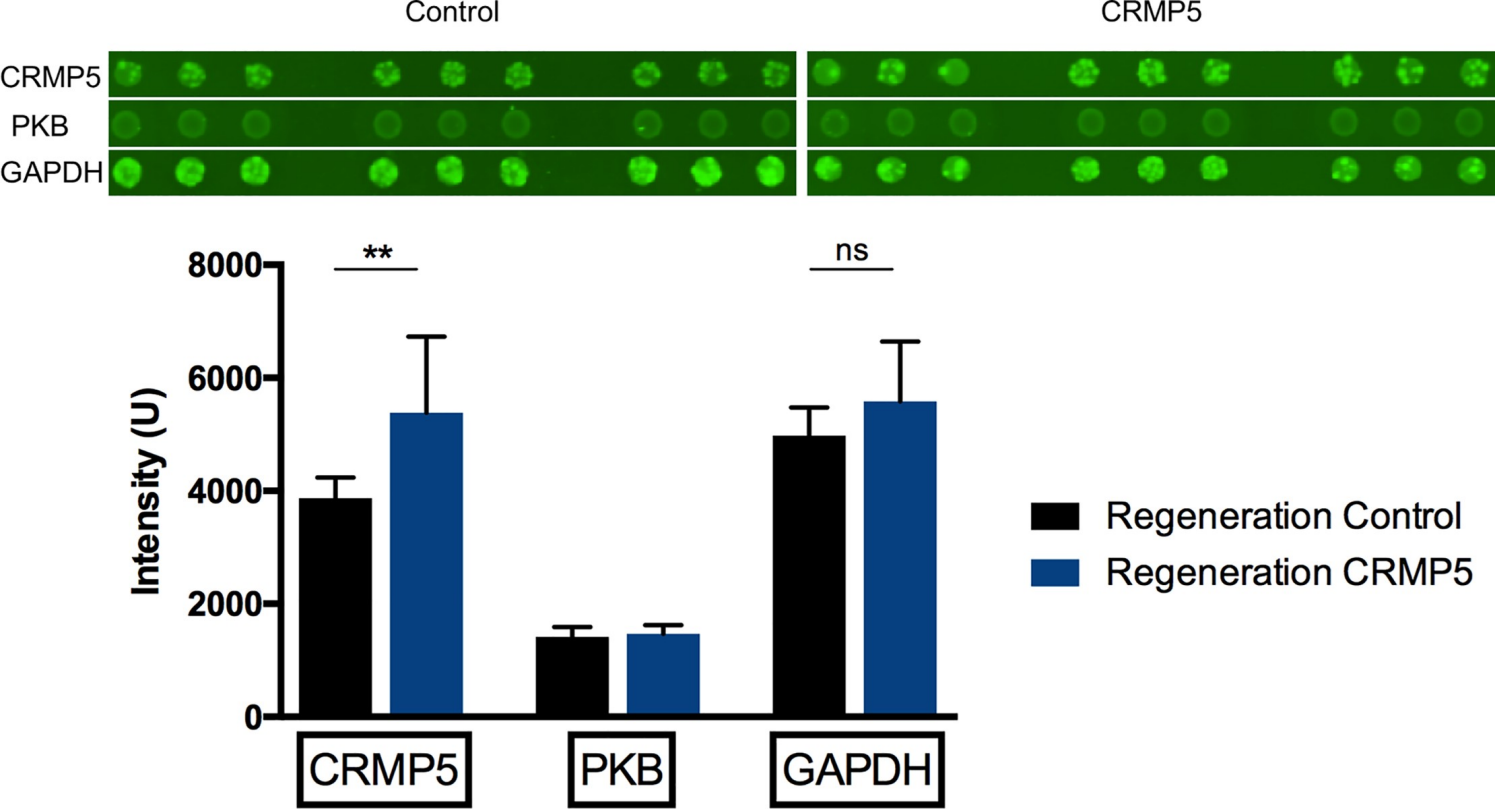
Detection of CRMP-5 and PKB in retinal proteome through microarray. CRMP-5 was detected significantly higher in the experimental group compared to the control group. Additionally, PKB was detected insignificantly higher in the experimental group. GAPDH served as loading control (n = 6, p < 0.01, ± SD, parametric t-test).

CRMP-5 was captured with an intensity of 3870 U within the control group (SD = 365.6) and 5382 U within the CRMP-5-displaced group (SD = 1350). PKB was captured with an intensity of 1414 U within the control group (SD = 178.5) and 1471 U within the CRMP-5-displaced group (SD = 154.6). GAPDH, as housekeeping protein, was detected with an intensity of 4978 within the control group (SD = 501.1) and 5585 U within the CRMP-5-displaced group (SD = 1057). Statistical analysis by a parametric t-test illustrated a significant increase in the intensity of CRMP-5 in the experimental group compared to the control group (p<0.01). PKB intensity was increased in the experimental group as well but without any significance (Fig 8).

### Assessment of the intracellular inclusion of CRMP-5 and the activation of PKB

**Fig 9:**
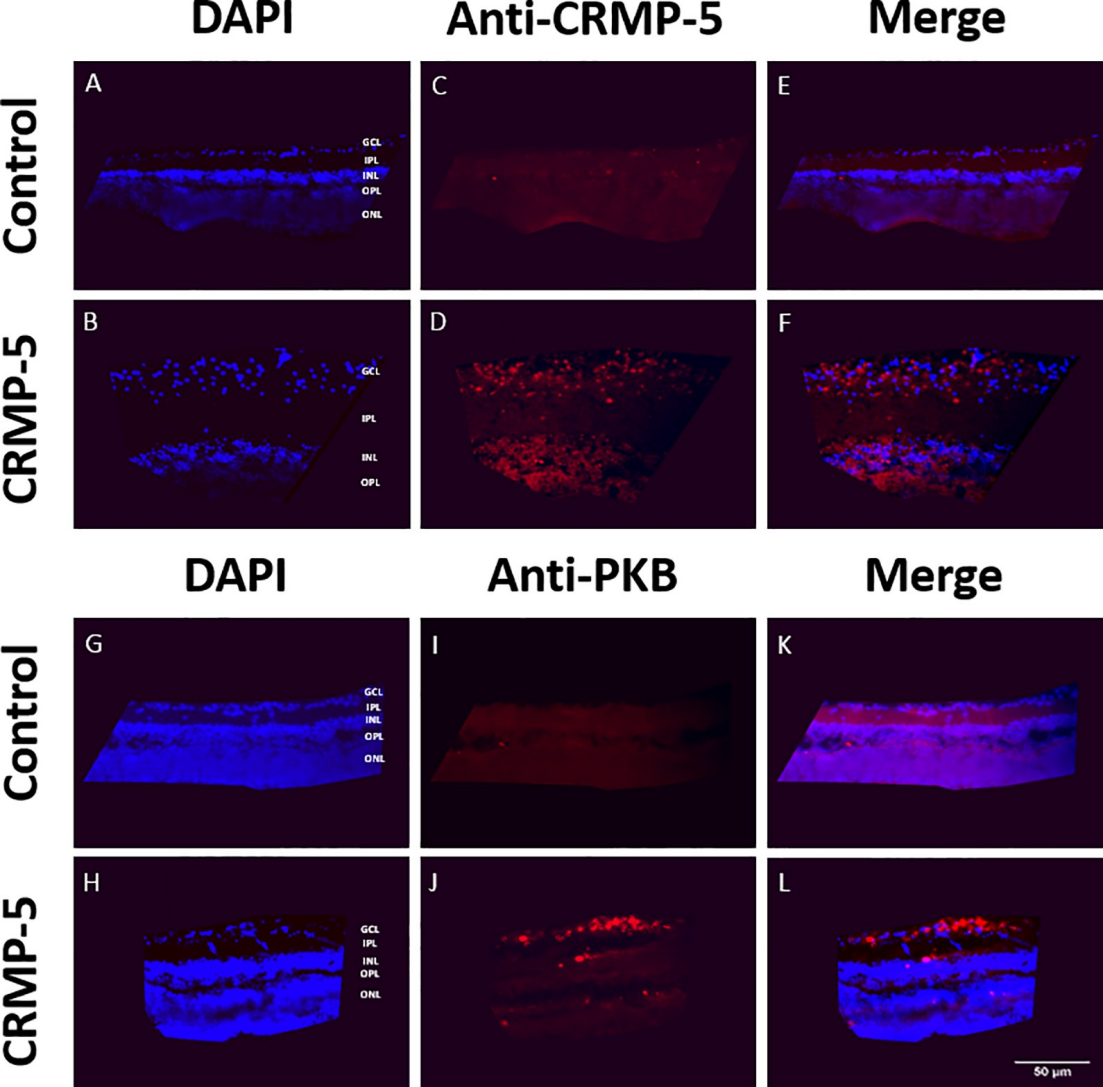
Immunohistochemical staining of retinal cross sections against CRMP-5 and PKB. Cross sections of retinal flatmounts, incubated with (B,D,F,H,J,L) or without (A,C,E,G,I,K) the addition of CRMP-5, were immunohistochemically stained against CRMP-5 (A-F) and PKB (G-L). CRMP-5 as well as activated PKB were detected in the ganglion cell layer of the explants, incubated with the addition of CRMP-5. **GCL** (Ganglion cell layer), **IPL** (inner plexiform layer), **INL** (inner nuclear layer), **OPL** (outer plexiform layer), **ONL** (outer nuclear layer). Scale bar 50 μm.

**Fig 10:**
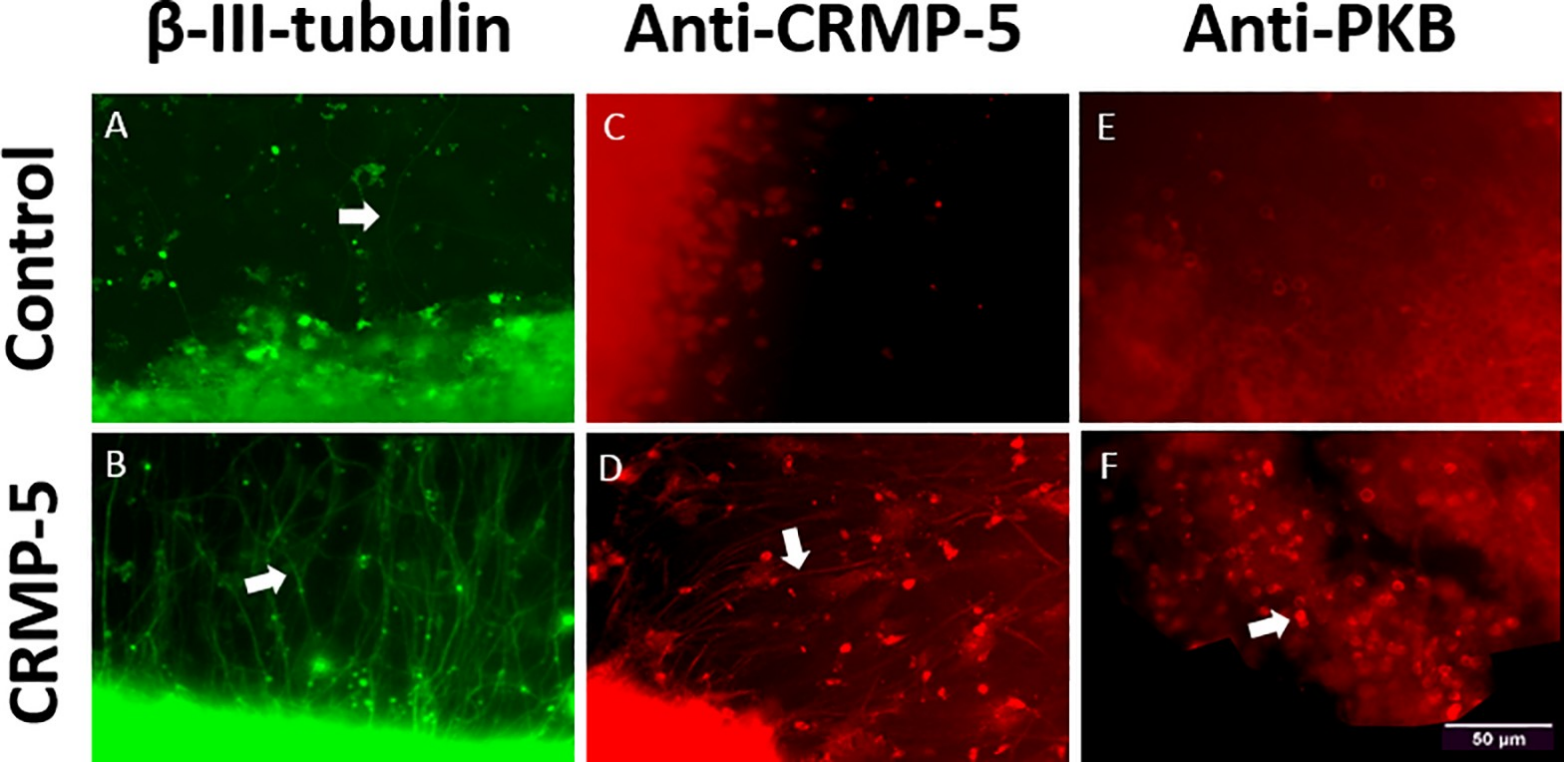
Immunohistochemical staining of retinal flatmounts against β-III-tubulin, CRMP-5 and PKB. Flatmounts of regenerating retinal explants incubated with (B,D,F) and without (A,C,E) the addition of CRMP-5, were stained immunohistochemically against β-III-tubulin (A,B), CRMP-5 (C,D) and PKB (E,F). The arrows point out outgrowing neurites within the control group (A) and the experimental group incubated with the addition of CRMP-5 (B,D). PKB was detected within the ganglion cells (F, Arrow points out RGC) and CRMP-5 in outgrowing neurites. Scale bar 50 μm.

Retinal cross-sections incubated with the addition of CRMP-5 featured a highly intense fluorescence after immunohistochemical staining against CRMP-5 or PKB. In both experimental groups especially, the ganglion cell layer was hallmarked by clearly demarcated and fluorescent cells (Fig 9). Retinal flatmounts stained against CRMP-5 after 7 days of incubation with the addition of CRMP-5 showed fluorescent outgrowing neurites. Fluorescence stretched over the entire length of neurites including growth cones whereas within the control group no neurites became visible. Retinal flatmounts stained against PKB after 7 days of incubation with the addition of CRMP-5 illustrated fluorescence intensification. Again, especially the ganglion cells seemed to be clearly demarcated and fluorescent (Fig 10).

## Discussion

This study exhibited three main findings. Firstly, a downregulation of CRMP-5 in glaucomatous retinal samples was detected. Secondly, CRMP-5 exerted neuroprotective as well as neuroregenerative effects on glaucoma-like injured RGCs. Thirdly, the effects of CRMP-5 on RGCs could be inhibited by an AB against PKB, exposing PKB as a possible downstream signaling pathway.

### Proteomic changes of glaucomatous rat retinas

In glaucomatous affected rat retinas CRMP-5 was detected three-fold downregulated via label-free mass spectrometry. It is supposed that the downregulation is caused by the increased IOP after episcleral vein occlusion. This gives reason to assume that CRMP-5 might be involved in the pathogenesis of glaucoma as it gets downregulated by an IOP elevation. CRMP-5 plays an important role in development and differentiation of CNS [9, 10] and is expressed in the embryonic and postnatal CNS [11, 18]. Except for brain regions running through processes of neurogenesis, CRMP-5 expression decreases in the adult CNS [12, 14, 18]. Hence in the adult retina CRMP-5 is found in low concentrations physiologically [18, 23] which even decrease through IOP elevation as shown in the present study. The elevated pressure causes apoptosis of the RGCs [24] with a combined decrease of CRMP-5 concentration in the adult retina.

### CRMP-5 promotes survival of glaucoma-like damaged RCGs

A high-pressure incubation chamber was utilized to mimic the physiological conditions of glaucoma in this *ex vivo* approach [25–27]. Compared to non-cultivated explants an incubation of the retinal organ culture reduced the number of RGCs. In turn, the addition of CRMP-5 affected an increase of the RGC-amount. Additional pressure decreased the number of vital RGCs even more but the application of CRMP-5 again increased it significantly. The immense gain of surviving RGCs after an addition of CRMP-5 lets us suggest, that CRMP-5 features neuroprotective properties. Cultivation as a stress factor for organ culture as well as pressure put on the RGCs could be diminished to a certain amount through CRMP-5. Underpinning these results, the application of denatured CRMP-5 showed no effect on the number of surviving RGCs. Again, pressure and cultivation caused apoptosis of RGCs [28–30]. This also confirms the hypothesis, that CRMP-5 promotes survival of the cells.

### Neuro-regenerative effects of CRMP-5 on RGCs

Our studies on neuro-regeneration point out that an application of CRMP-5 to the culture medium, prior to cultivation, leads to a significant elongation as well as an increased number of outgrowing neurites. CRMP-5 plays an important role in CNS development [9, 10]. It is expressed mainly in neuronal structures [15] of the embryonic and early post-natal CNS [11], more specifically in post-mitotic precursors and is essential for neuronal differentiation [13]. However, albeit in small quantities, it is detectable in adult organisms [13, 31]. Hence, the outgrowth stimulation and elongation of the neurites in this study, triggered by the addition of CRMP-5, can be led back to its regeneration promoting effects. RGCs were stimulated to regeneration through preliminary ONC and LI [32–34]. The injury of the RGC axons imitates the pathophysiological effect of increased IOP to the retina and transfers RGCs into a condition enabling regeneration [35–38]. LI also induces neuroprotective and neuroregenerative effects *in vivo* and *in vitro* [39, 40]. These effects are mediated e.g. through lentiginous crystallines [39, 41, 42], that cause an increase in neurite outgrowth [40, 41, 43, 44]. After the application of CRMP-5, RGCs generated not only a higher number of outgrowing neurites but especially longer ones. Thus, the admission of the protein puts additional RGCs into a regeneration able state. Studies on the expression of different CRMPs in neurons of the olfactory bulb after axotomy, showed an expression of CRMP-5 in immature regenerating neurons whereas in adult degenerating neurons only high concentrations of other CRMPs, like CRMP-1 and -2 were detected [18]. Studies on CRMP-5 knock-out mice featured diminished cell bodies of cerebellar Purkinje cells with smaller dendritic calibers [45]. These findings might hint at the fact that RGCs (as neurons of the CNS) might show similar cell body changes once CRMP-5 is lacking.

Furthermore, the uptake of the protein caused acceleration of neurite outgrowth, attributed to growth promotion of cone guidance, neuronal polarity and longitudinal growth by CRMP-5. [14, 15, 18].

The classification of outgrowing neurites grouped by their lengths shows that especially the number of medium and long neurites increased tremendously through addition of CRMP-5 compared to the control group. Correspondingly the addition of CRMP-5 seems to cause an accelerated neurite lengthening. Seven days PE with the addition of CRMP-5 the number of short and medium neurites increased by half compared to day five PE whereas long neurites doubled their amount. On the one hand new neurites are built during this period, which increase the total amount. On the other hand the progress of cultivation time in addition to the application of CRMP-5 lead to an acceleration of neurite outgrowth. Thus, the longer retinal explants are cultivated the better CRMP-5 can unfold its impact and the faster neurite outgrowth gets.

### CRMP-5 mediates neuroprotective and neuroregenerative effects through an interaction with PKB

Neuroprotective effects of CRMP-5 might result from an interaction with PKB (9). This could be shown via microarray and immunohistochemistry within the neuroprotection studies as well as neuroregeneration studies. Increased levels of intracellular CRMP-5 as well as PKB could be detected within the samples incubated with an addition of CRMP-5 via microarray. This suggests a correlation between the uptake of CRMP-5 and the activation of PKB. What underlines this fact is the allocation of fluorescence in the immunohistochemical stainings. It shows an activation of PKB and a detection of CRMP-5 limited to the RGCs whereas CRMP-5 was also visualized in outgrowing neurites.

Various correlations between CRMP-5 and an activation of PKB are discussed. On the one hand CRMP-5 was detected in high concentrations in numerous neoplastic tissues such as breast, lung, prostate, or pancreatic tumors [9, 10]. It is considered a marker for lung carcinoma and increases the proliferation rate of glioblastoma [9, 46]. Presumably CRMP-5 controls notch-signaling in glioblastoma through a protection of notch-receptors against lysosomal degradation [9]. This signaling pathway is linked to the activation of PKB and hence to proliferation of the tumor [9, 47, 48]. On the other hand, the increased activation of PKB might be due to an interaction between CRMP-5 and brain derived neurotrophic factor (BDNF). An involvement of CRMP-5 in the BDNF signaling pathway is assumed as in CRMP-5 knock-out mice the BDNF effect is notably low. In Purkinje cells CRMP-5 is phosphorylated by Tyrosin-kinase B (TrkB) and thus regulates their dendritic morphology [45, 49]. Through extracellular signals, like e.g. growth factors, the receptor tyrosine kinase (RTK) activates Phosphoinositide 3-kinase (PI3K). PI3K initiates the PI3K/Akt- signaling pathway through generation of the secondary messenger Phosphatidylinositol (3,4,5)-trisphosphate (PIP3) out of Phosphatidylinositol (4,5)-bisphosphate (PIP2). Thereupon PKB with its pH-domain binds to PIP3 where it gets phosphorylated at its amino acids Serine(473) and Threonine(308) [17]. The iatrogenic induced spillover of CRMP-5 in this study, which probably gets phosphorylated by TrkB, leads to a better signal transduction of BDNF through it. This results in an increased activation of PKB and its anti-apoptotic effects.

PKB is essential for the regulation of different intracellular processes such as cell growth and proliferation. PKBs belong to a group of Serine/Threonine kinases and are expressed in all kinds of tissues. Composed of three enzymes, PKB-α, -β and –γ they transfer a phosphate group to other proteins. Hence, they represent an essential component of anti-apoptotic cell signaling pathways by changing activity of transcription factors. Furthermore, it operates as an oncogene as it is dysregulated in several neoplastic diseases. At this juncture the Phosphatase and Tensin homolog (PTEN) is inactivated by mutation and does not inhibit PIP3. The resultant overactive PKB induces an increased cell proliferation [9, 16, 17, 50, 51].

**Fig 11:**
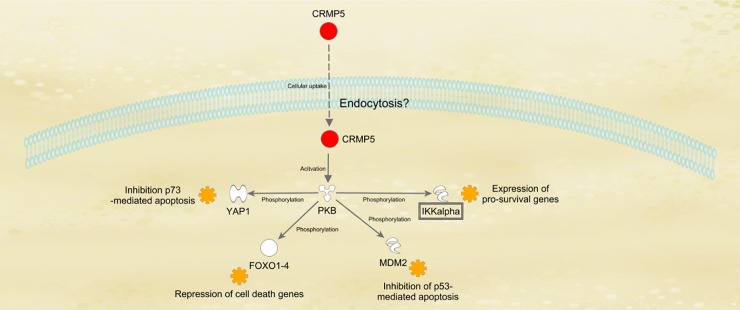
The potential neuroprotective mechanism of CRMP-5 (Ingenuity).

The usage of an antibody against activated PKB within our studies on neuroprotection nearly completely reversed the protective effects of CRMP-5. This result also speaks for an interaction between CRMP-5 and PKB or rather an activation of PKB through CRMP-5 [9]. By phosphorylating for instance transcription factors, such as Yes-associated-protein 1 (YAP1) [52], Forkhead box protein 1–4 (FOXO 1–4) [53], Mouse double minute 2 homolog (MDM2) [54] and I kappa b kinase [55] activated PKB triggers different anti-apoptotic signaling pathways. These contain the suppression of pro-apoptotic genes, the inhibition of p53- [56] or p73-mediated apoptosis and the expression of cell survival genes [16]. These effects were absent after the application of the AB against activated PKB (Fig 11).

## Conclusion

CRMP-5 seems to play an important pathophysiological role in an animal model of glaucoma and exerts both neuroprotective and neuroregenerative effects *in vitro*. We suspect the down-regulation as a reaction on the IOP elevation. Application of CRMP-5 could enhance the RGC survival tremendously. Furthermore, it capacitated RGCs to a higher regeneration rate and to an accelerated neurite outgrowth. This effect could be possibly mediated by an activation of Protein Kinase B. PKB affects many intracellular pathways such as inhibition of apoptosis or expression of pro-survival genes.

## Abbreviations

AB: Antibody
CRMP-5: Collapsin-Response-Mediator-Protein-5
IOP: Intraocular pressure
LFQI: Label-free quantification intensity
LI: Lense injury
ONC: Optic nerve crush
OD: Oculus dexter
OS: Oculus sinister
PBS: Phosphate-buffered saline
PE: Post explantationem
PKB: Proteinkinase B
RGCs: Retinal ganglion cells
SD: Sprague Dawley
